# Experimental dataset on the compressive mechanical properties of high-density pressboard used in power transformers spacers

**DOI:** 10.1016/j.dib.2023.109471

**Published:** 2023-08-03

**Authors:** Carmela Oria, Isidro Carrascal, Diego Ferreño, Cristina Méndez, Alfredo Ortiz

**Affiliations:** a Electrical and Energy Department, Universidad de Cantabria, Spain; b Laboratory of Science and Engineering of Materials, Universidad de Cantabria Avenida, Los Castros, s/n, Santander 39005, Spain

**Keywords:** Cellulosic insulation of power transformers, Mechanical properties of pressboard, Mechanical testing of pressboard, High-density pressboard, Thermal ageing

## Abstract

The structural elements within power transformers are commonly constructed from high-density pressboard, chosen for its favourable mechanical and dielectric properties. Among these elements are the spacers employed in the windings of transformers, which endure compressive loading during operation. The spacers are immersed in dielectric fluid and exposed to high temperatures and chemical reactions over the transformer's lifespan, resulting in the degradation of their dielectric and mechanical properties. The mechanical integrity of the power transformer significantly relies on these factors; hence, it is imperative to comprehend how ageing deteriorates the mechanical response of the high-density pressboard.

The present article presents experimental data on the compressive mechanical properties of a commercially available high-density pressboard, commonly employed in power transformer spacers, under various ageing conditions (induced through accelerated thermal ageing and assessed by the degree of polymerisation). These data hold potential for diverse applications. They can enhance the existing comprehension of the mechanical behaviour and degradation mechanisms of cellulosic insulation in power transformers and provide reference benchmarks for comparison with factory-obtained values by manufacturers. In the realm of engineering failure analysis, these values can be utilised to evaluate the mechanical failures of paper-based materials utilised as structural components in power transformers.

Specifications Table

**Table:** 

Subject	Mechanics of Materials
Specific subject area	Characterisation of the compressive mechanical properties of high-density pressboard employed in power transformer spacers
Type of data	Table Graph Figure
How the data were acquired	The experimental dataset was obtained by employing the average viscometric degree of polymerisation, as per the guidelines outlined in IEC 60450 [1]. To test the high-density pressboard under compressive loading, adaptations were made to standards [2,3]. For a comprehensive understanding of the experimental tests' detailed design, please refer to Section 2*.*
Data format	Raw Analysed
Description of data collection	Samples of high-density pressboard, specifically used as spacers in power transformers, were prepared with dimensions suitable for compressive mechanical testing. These samples were impregnated with paraffinic dielectric oil and placed inside sealed vessels along with copper pieces, mimicking the pressboard-to-copper ratio found in transformer windings. Subsequently, the samples were subjected to ageing in convective ovens at 150 °C for varying durations. To assess the effects of ageing, the degree of polymerisation was measured in both new and aged pressboard samples, following the guidelines outlined in IEC 60450. Square pressboard samples, with dimensions of 30 mm on each side, were arranged in a stacked configuration to create a prism-shaped specimen for conducting compressive tests. The prism was subjected to three cycles of loading and unloading using a servo-hydraulic universal testing machine. Throughout the loading cycles, the servo-hydraulic testing machine applied a gradually increasing load of up to 4 kN at a constant rate of 0.1 kN/s. The compressive load (N) was accurately recorded using a load cell, while the deformation of the pressboard prism (mm) was measured by tracking the displacement of the hydraulic actuator.
Data source location	The high-density pressboard was provided by Imefy. The experimental testing was carried out at the Electrical and Energy Department, and at the Laboratory of Science and Engineering of Materials (University of Cantabria), in Santander, Cantabria, SPAIN.
Data accessibility	https://data.mendeley.com/datasets/gdt2xwrbtz/3
Related research article	C. Oria, C. Méndez, I. Carrascal, D. Ferreño, and A. Ortiz Degradation of the compression strength of spacers made of high-density pressboard used in power transformers under the influence of thermal ageing. Cellulose, 2023. DOI: 10.1007/s10570-023-05268-8

## 1 Value of the Data

• The electrical, chemical, and mechanical degradation of the cellulosic insulation components are widely acknowledged as the primary factors leading to the end-of-life of transformers, resulting in failures through various mechanisms [4]. The majority of the components within power transformers, serving both structural and dielectric functions, are constructed using high-density pressboard. One such component is the radial spacer, which experiences predominantly compressive loading in the trough-thickness direction. In this study, we present data concerning the compressive loading response of the high-density pressboard under various ageing conditions.
• Presently, the majority of models used for predicting the end-of-life of cellulosic insulation in power transformers rely on the degree of polymerisation (DP). Nevertheless, it has been demonstrated that this approach is not suitable in many cases, as the material maintains its mechanical integrity even at very low DP values (refer to Ref. [5]). To effectively manage power transformers throughout their operational life, a deeper understanding of the evolution of mechanical properties is essential.
• There is a lack of comprehensive literature documenting the compressive mechanical behaviour in the trough-thickness direction of high-density pressboard under realistic operating conditions in power transformers. This knowledge gap has posed challenges in comprehending the deterioration of mechanical integrity observed in spacers within core-type power transformers. To address this issue, it is crucial to develop reliable techniques capable of identifying loose clamping conditions within transformer windings. These conditions can arise due to compressive loading induced by axial electromagnetic forces or other mechanical loads experienced during the transformer's operational lifespan, across various ageing levels. Paper manufacturers, power transformer manufacturers and researchers working in the field of paper mechanics can benefit from the presented data.
• The data presented in this study provides valuable reference values for the quality control of manufacturing processes within the paper industry, particularly concerning high-density pressboard with similar grammage and thickness to the material studied. By using these reference values, the assessment and enhancement of cellulosic insulation materials with improved mechanical properties can be facilitated.
• Additionally, the data presented in conjunction with the described experimental design can be employed by electric utility companies responsible for managing power transformers within electric systems. This application enables the diagnosis of the mechanical performance of components crafted from high-density pressboard within operating transformers, particularly in ageing states comparable to those studied in this research. These findings have significant implications for establishing end-of-life criteria for transformers, facilitating proactive maintenance and replacement strategies.
• For researchers in the field of power transformers, material properties play a crucial role in developing numerical models using FE software to obtain reliable results. Typically, the properties of cellulosic insulation are acquired through expensive and time-consuming accelerated thermal ageing in laboratories. Open access to trustworthy material properties streamlines the development of these numerical models.

## 2 Objective

The objective of this data article is to present a comprehensive dataset that captures the compressive mechanical properties of high-density pressboard utilised in spacers within power transformers. The dataset was generated through a series of experiments conducted under different ageing states induced by accelerated thermal ageing. The degree of polymerisation was employed to quantify the ageing levels. By providing this dataset, we aim to contribute to the existing body of knowledge regarding the mechanical response and degradation processes of cellulosic insulation in power transformers. The dataset can serve as a valuable resource for researchers and engineers involved in the development and assessment of insulation materials with improved mechanical properties during the manufacturing process.

Moreover, this dataset provides a valuable supplement to the corresponding original research article, referenced as Ref. [6], as it offers empirical evidence and precise quantitative measurements of the compressive loading curves. These curves serve as the basis for the stiffness values reported in the referenced article. It offers a reliable reference point for comparing similar datasets obtained by manufacturers in factory settings. In summary, this data article aims to provide a comprehensive dataset that enhances the understanding of the mechanical behaviour and ageing effects on high-density pressboard, thereby contributing to the improvement of power transformer design, performance, and reliability.

## 3 Data Description

**Graph 1:**
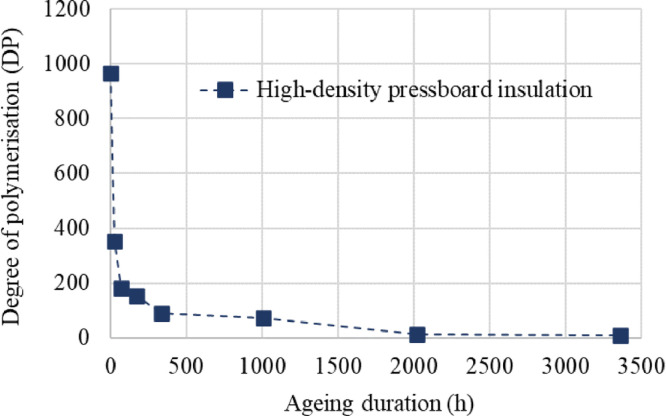
Degradation of degree of polymerisation in high-density pressboard with increasing duration of thermal ageing.

Table 1 presents the manufacturing properties of the high-density pressboard supplied by Imefy and the properties of the paraffinic oil used for ageing the pressboard. The degree of polymerisation (DP) of the pressboard, as influenced by the ageing duration, is illustrated in Graph 1.

**Table 1:** Manufacturing properties of the high-density pressboard and the paraffinic dielectric oil used in the experiments. Adapted from Ref. [6].

High-density pressboard			Paraffinic dielectric oil		
Property	Unit	Value	Property	Unit	Value
Thermal class	°C	105	Density at 20 °C	g/cm^3^	0.839
Apparent density	g/cm^3^	1.2	Viscosity at 40 °C	mm^2^/s	9.98
Nominal Thickness	mm	2	Viscosity at -30 °C	mm^2^/s	925.85
Tensile Strength (MD)	MPa	124	Flash point	°C	176
Tensile Strength (CD)	MPa	92	Pour point	°C	-48
Elongation at breakage (MD)	%	3.9	Interfacial tension	mN/m	43
Elongation at breakage (CD)	%	4.6	Dielectric dissipation factor, 90 °C	–	0.00198
Compressibility	%	4.8			
Shrinkage (ZD)	%	4.4	Acidity	mg KOH/g	<0.01
Shrinkage (MD)	%	0.4	Water content	mg/kg	15
Shrinkage (CD)	%	0.5	Furfural content	mg/kg	<0.05
Oil absorption	%	13

**Fig. 1:**
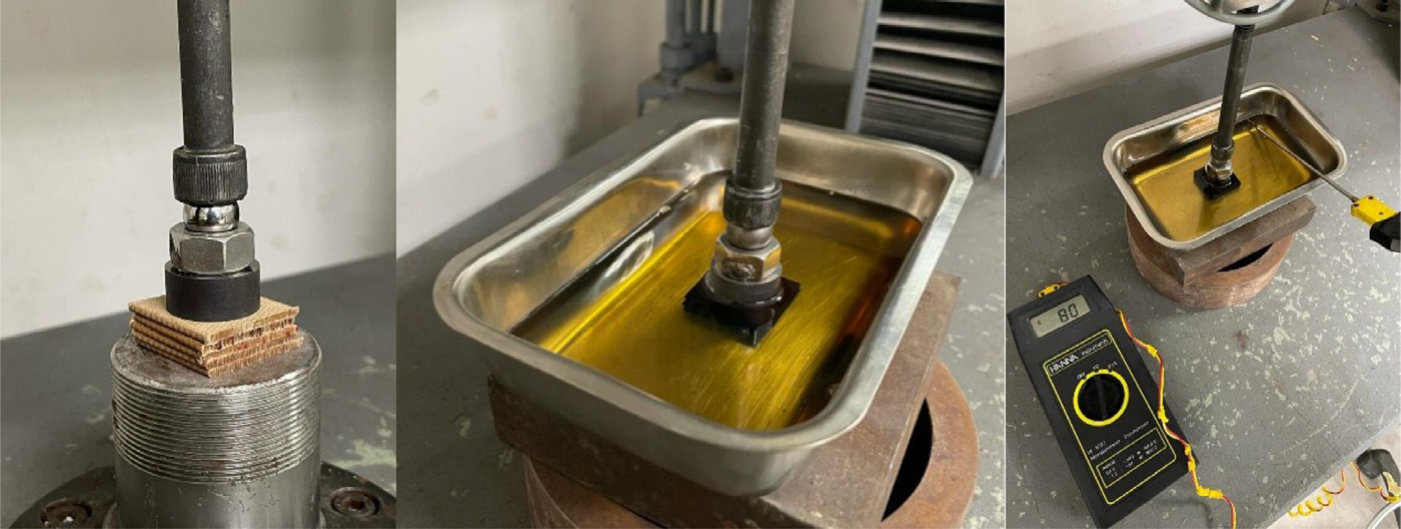
Compressive testing of high-density pressboard prism: Open-air condition and immersed in dielectric oil. Adapted from Ref. [6].

**Graph 2:**
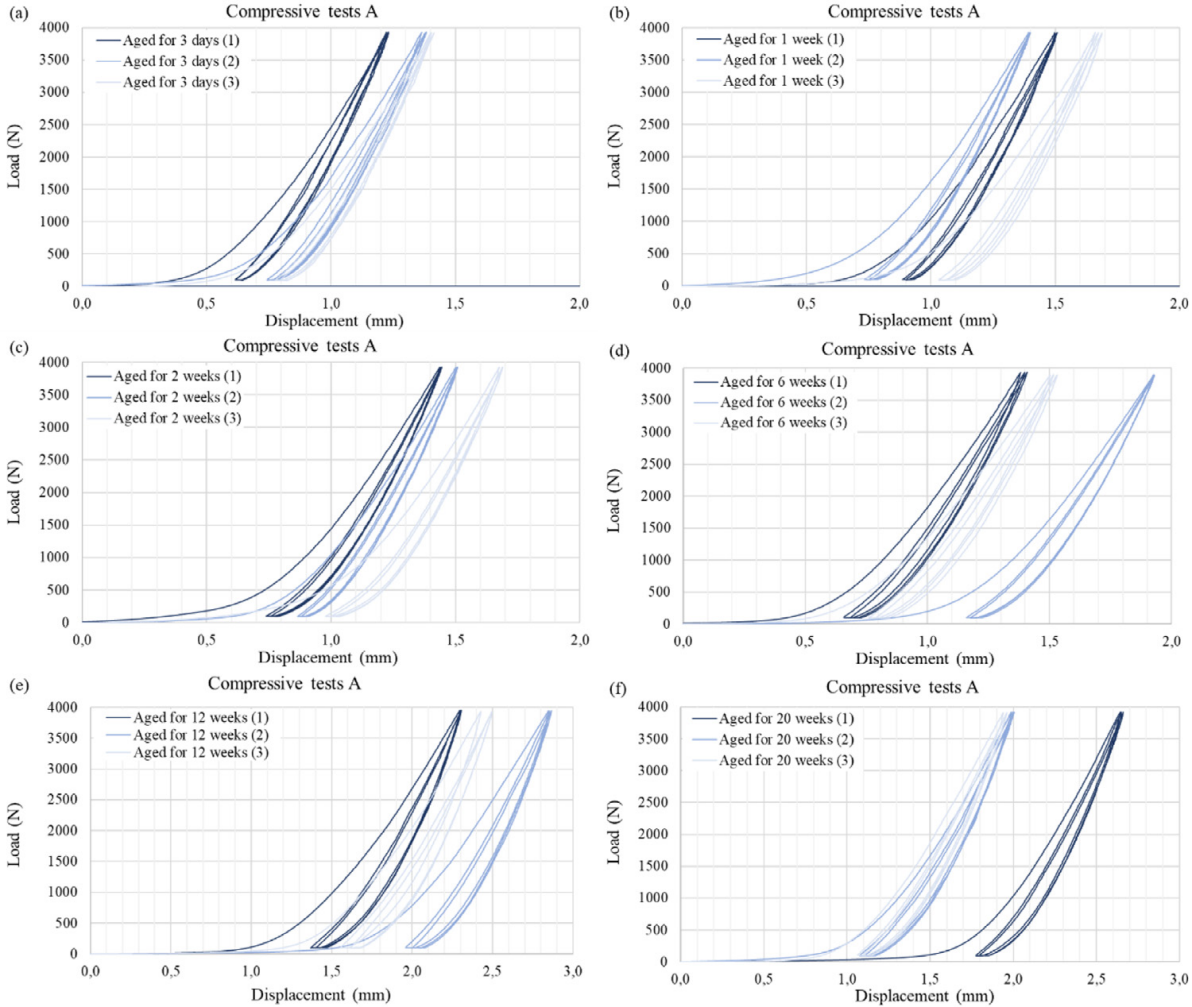
Compressive Test A: Load-displacement curves of the pressboard prism with three loading-unloading cycles at various ageing durations (a) 3 days, (b) 1 week, (c) 2 weeks, (d) 6 weeks, (e) 12 weeks and (f) 20 weeks. Adapted from Ref. [6].

**Graph 3:**
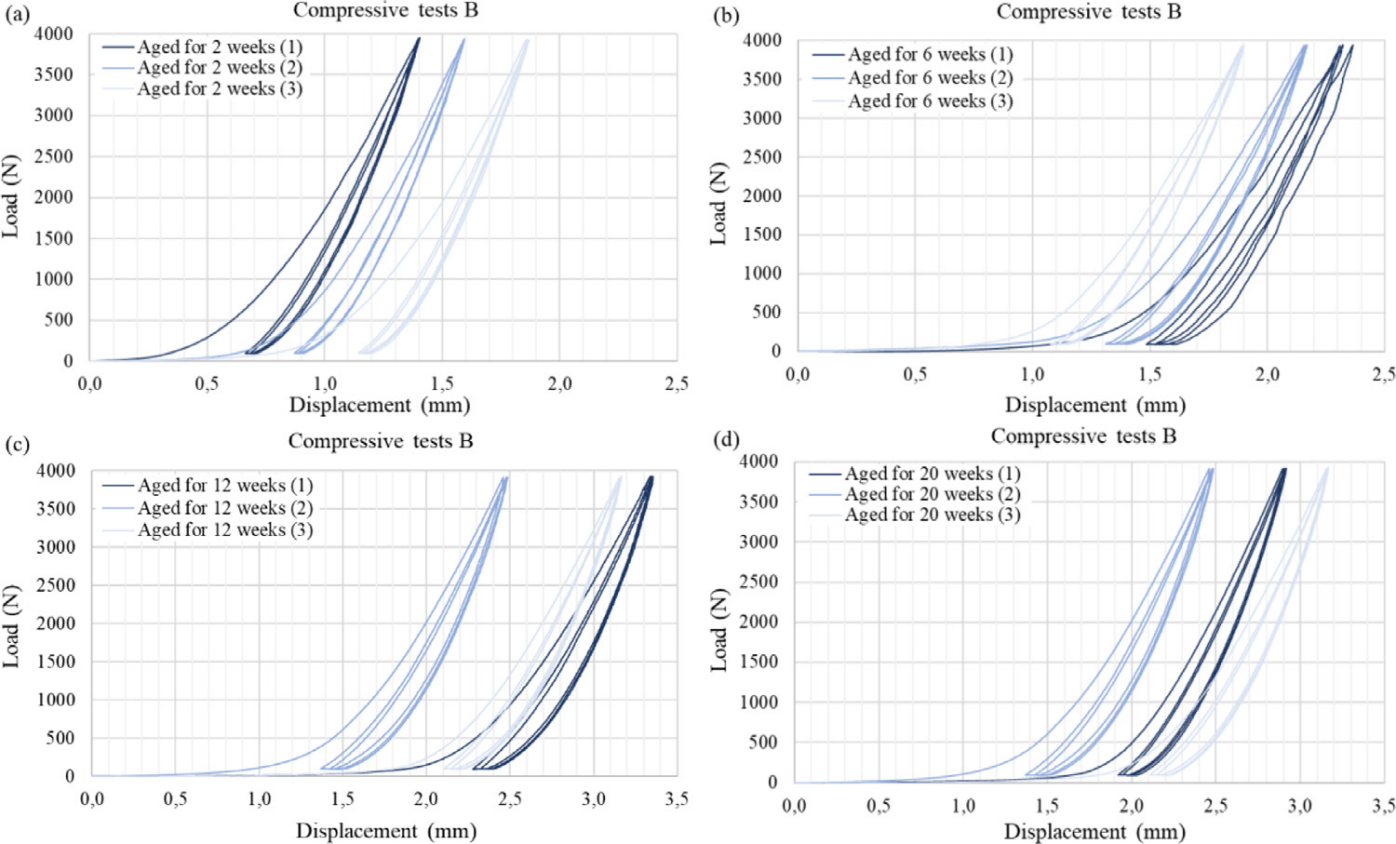
Compressive Test B: Load-displacement curves of the pressboard prism with three loading-unloading cycles at various ageing durations (a) 2 weeks, (b) 6 weeks, (c) 12 weeks and (d) 20 weeks. Adapted from Ref. [6].

Fig. 1 displays the configuration of the prism samples used for compressive testing in two distinct setups: open-air and immersed in oil at 80 °C. Graphs 2 and 3 depict the compressive force-displacement curves during loading and unloading cycles for the pressboard prism in various ageing conditions.

## 4 Experimental Design, Materials and Methods

### 4.1 Accelerated Thermal Ageing of the High-Density Pressboard

In this study, square samples with a side length of 30 mm were prepared from high-density pressboard spacers, whose manufacturing properties are specified in Table 1. These samples were then placed in an oven and dried for a period of 4 hours at a temperature of 105 °C. The final moisture content, measured according to Ref. [7], was found to be 1% after the drying process. The paraffinic oil used, whose properties are listed in Table 1, was also subjected to vacuum drying at 105 °C for 3 h to reduce its initial moisture content to 32.1 ppm. Moisture was analysed using an automatic coulometer (model 899, manufactured by Metrohm), equipped with an integral magnetic stirrer. It enables the measurement of water contents ranging from 10 µg to 200 mg.

After the initial drying and oil impregnation, the pressboard samples were divided into groups of 45 samples, with a total mass of approximately 95 g. These samples were then placed in a stainless-steel vessel containing 700 ml of mineral oil, along with copper conductor pieces weighing a total of 1340 g. The proportion of pressboard to copper in the vessel was maintained to reflect a similar ratio found in power transformer windings, considering any potential catalytic effects of copper on the pressboard aging process.

Prior to filling the vessels with nitrogen to establish an inert atmosphere, they were vacuum-sealed using a Telstar pump (model Top-3). The nitrogen, sourced from Air Liquide, possessed extremely low levels of impurities, with content specifications not exceeding 3 ppm of H_2_O, 2 ppm of O_2_, and 0.5 ppm of CnHn. The pressure within the vessels was regulated through a pressure gauge. Subsequently, the vessels were placed in temperature-controlled ovens and aged at 150 °C for various durations: 3 days, 1 week, 2 weeks, 6 weeks, 12 weeks and 20 weeks. The Memmert UF 110 stove operates under forced air circulation, with a temperature range from +20 °C to +300 °C. It is equipped with a 1 Pt100 sensor (DIN Class A) using a 4-wire measurement system. Some samples were kept unaged to serve as the initial condition of the insulation material.

The ageing temperature of 150 °C was selected for this study based on prior publications by the same authors involving diverse cellulosic materials used as insulation in transformers [8], [9], [10]. These earlier investigations revealed that a lower ageing temperature would have required over seven months of accelerated thermal ageing to achieve an insulation state comparable to the entire operational lifespan of an actual transformer.

The degree of polymerisation (DP) was determined in accordance with Ref. [11], following the removal of residual oil from the aged high-density pressboard using distilled hexane. Subsequently, a sample of the pressboard was fragmented and dissolved in a solution comprising 22.5 g of deionised water and 24.75 g of cupriethylenediamine hydroxide. The resulting solution was subjected to magnetic stirring for 16 h, with the vial containing glass balls to prevent moisture absorption. The kinematic viscosity of the solution was assessed subsequently, serving as an indicator of the material's molecular weight, employing an Ubbelohde viscometer with a constant of 0.011269. The temperature was consistently regulated at 20 °C throughout the measurements through the use of a thermostatic water bath provided by PolyScience. The experimental temperatures spanned from +10 °C to 200 °C, exhibiting a remarkable temperature stability of ±0.005 °C. The results of the DP measurements are presented in Graph 1.

## 5 Compressive Mechanical Properties of the Aged High-Density Pressboard

At present, there is a lack of universally accepted standards specifically designed for measuring the compressive properties of pressboard used in electrical applications. However, several publications, including Refs. [12,13], have explored the variability of compressibility in high-density pressboard by examining the relationship between compressive strain (%) and specific compressive stress (MPa) under various testing conditions, such as ageing, temperature, or moisture content. In contrast, existing standards for other materials, such as concrete and polymers (see Refs. [2,3]), primarily emphasise the importance of compressive stiffness as the key parameter for characterising the compressive mechanical response. For pressboard, compressive stiffness signifies its ability to withstand deformations in the thickness direction when subjected to compressive loads. Higher stiffness indicates reduced deformations in components fabricated from high-density pressboard, consequently enhancing the overall mechanical integrity of the power transformer. Hence, it is imperative to investigate the variations in compressive stiffness with respect to ageing duration and evaluate whether the presence of dielectric oil at specific temperatures affects its value. Gaining insights into these dynamics will advance our understanding of the mechanical behaviour of high-density pressboard and its implications for the performance of power transformers.

Due to its thinness of only 2 mm, measuring deformations directly on a single square sample of pressboard during compressive tests is impractical. To enable more precise and representative measurements by accommodating larger deformations, a stack of five square pressboard samples, each with a side length of 30 mm, was assembled to form a prism, see Fig. 1. This prism was subjected to three cycles of loading and unloading, using a Servo-hydraulic Universal testing machine (manufactured by SERVOSIS, model ME-405/01), equipped with a ±5 kN capacity load cell and ±50 mm stroke actuator, operating under quasi-static displacement control conditions. To ensure perpendicular loading on the pressboard faces, a 24 mm diameter steel loading plate with a ball joint was used for load transfer. During the loading cycles, the servo-hydraulic testing machine gradually increased the load up to 4 kN at a speed of 0.1 kN/s, simultaneously recording the compressive load (N) through the load cell and the deformation of the pressboard sample (mm) *via* the displacement of the hydraulic actuator. The compressive stress was calculated using Eq. (1), where, F represents the compressive load (in N). In the conducted tests, the maximum compressive stress reached 8.84 MPa, a value typically observed as clamping pressure in power transformers according to Ref. [12]. The deformation experienced by the compressed prism is the sum of the deformations endured by each of the five square pressboard samples. It can be assumed that the deformation of each pressboard sample is approximately equal, thereby enabling the definition of the material's stiffness, $K_{\text{pressboard}}$, as five times the stiffness of the tested pressboard prism, $K_{\text{prism}}$, as shown in Eq. (2).

(1) $$\sigma =\frac{F}{\pi 12^{2}}\;\left(MPa\right)$$

(2) $$K_{pressboard}=5\cdot K_{prism}\;\left(N/mm\right)$$

In this study, two types of compressive tests were conducted on prisms fabricated from aged high-density pressboard, see Fig. 1. The first type, referred to as ``Tests A'' henceforth, involved cleaning the aged pressboard samples using distilled hexane to eliminate any residual dielectric oil remaining from the thermal ageing process. Subsequently, compression was performed under open-air conditions at room temperature. In the second type, designated as ``Tests B,'' the aged pressboard samples were subjected to compression while immersed in the same paraffinic dielectric oil used during the ageing process. The compression took place at a temperature of 80 °C, which is commonly encountered in operational power transformers and represents a typical operating condition.

For each ageing period considered, a series of three compressive tests were conducted on the pressboard prism to obtain average values for the material's compressive stiffness which are presented in Ref. [6]. Pressboard, being a cellulosic polymer with a porous structure, exhibits a strongly non-linear behaviour in the thickness direction (ZD), see Graphs 2 and 3. The force-displacement curves display two distinct regions. Initially, during the first loading cycle, a notable increase in compressive deformation is observed with only a slight increase in compressive force. This response is attributed to the porous nature of the material, wherein the porous structure collapses as it undergoes compression in the ZD, resulting in plastic deformation. Subsequently, a region follows where the curve's slope steepens, requiring greater force to induce further compressive deformation. This change is attributed to the combined effects of increased elastic stiffness and plastic hardening [12].

Figs. 10 and 11 from the related research article, see Ref. [6] only depict the third loading-unloading cycle for visual clarity, because compressive stiffness of the high-density pressboard was derived from this particular cycle. However, in this study, we present the complete set of compressive tests in Graphs 2 and 3. These comprehensive tests provide valuable insights into the densification process and its impact on this specific material. Additionally, the accompanying data for this article, including the CSV files containing load-displacement values for each test, enable the calculation of other pertinent mechanical parameters that describe the response of the high-density pressboard to compressive loading. It is worth noting that, as discussed in the related research article, there are no existing references for obtaining the compressive stiffness of pressboard used for electric applications. Therefore, in our study, the compressive stiffness was defined based on different load levels (refer to Table 4 in Ref. [6]). However, it should be acknowledged that alternative definitions derived from the experimental data are also possible.

## Ethics Statement

The authors have read and follow the ethical requirements for publication in Data in Brief. The current work does not involve human subjects, animal experiments, or any data collected from social media platforms.

## CRediT authorship contribution statement

**Carmela Oria:** Formal analysis, Writing – original draft, Writing – review & editing, Visualization. **Isidro Carrascal:** Methodology, Investigation. **Diego Ferreño:** Conceptualization, Methodology, Writing – review & editing, Supervision. **Cristina Méndez:** Methodology, Investigation. **Alfredo Ortiz:** Conceptualization, Methodology, Project administration.

## Data Availability

Experimental Dataset on the Compressive Mechanical Properties of High-density Pressboard Used in Power Transformers Spacers (Original data) (Mendeley Data).
