# Corrigendum to “Experimental dataset on the compressive mechanical properties of high-density pressboard used in power transformers spacers” [Data in Brief 50 (2023) 109471]

**DOI:** 10.1016/j.dib.2023.109498

**Published:** 2023-08-16

**Authors:** Carmela Oria, Isidro Carrascal, Diego Ferreño, Cristina Méndez, Alfredo Ortiz

**Affiliations:** a Electrical and Energy Department, Universidad de Cantabria, Spain; b Laboratory of Science and Engineering of Materials, Universidad de Cantabria Avenida, Los Castros, s/n, Santander 39005, Spain

The authors regret an error in the acknowledgments of the original manuscript, which has been corrected in the present corrigendum:

The authors gratefully acknowledge funding for this work from the *Ministerio de Ciencia e Innovación*, under the grant agreement PID2019-107126RB-C22. Additionally, we would like to express our gratitude to Imefy for providing the high-density pressboard, the mechanical properties of which are reported in the present article.

The authors would like to apologise for any inconvenience caused.

